# Improving the Retention of Low-Volume Autologous Fat Grafting: A Comparative Analysis of Lipoaspirate Processing Techniques for Facial Feminization

**DOI:** 10.1093/asjof/ojae043

**Published:** 2024-06-03

**Authors:** Katherine H Carruthers, William G Austen, Katya Remy, Ryoko Hamaguchi, Sofia Liu, Krishna Vyas, Branko Bojovic

## Abstract

**Background:**

Outcomes following autologous fat grafting have historically been unpredictable because of variability in fat retention rates. The novel poloxamer wash, absorption, mesh filtration system (PWAS) uses proprietary technology to stabilize and concentrate lipoaspirate. Its use in low-volume fat grafting has not been reported.

**Objectives:**

The authors in this study aimed to compare PWAS technology with traditional lipoaspirate processing techniques in low-volume fat grafting procedures.

**Methods:**

Medical charts were reviewed to determine a consecutive cohort of patients who underwent fat grafting for facial feminization. All patients had obtained preoperative and postoperative 3-dimensional facial imaging. Patients were grouped based on the method of lipoaspirate processing. The analysis software was used to measure changes in facial volume, and percent retention was calculated.

**Results:**

Between September 2021 and February 2023, 11 facial fat grafting procedures were performed using the PWAS, and 5 performed using traditional lipoaspirate osmotic filtration with Telfa. Age and BMI were statistically similar between both the groups (*P* > .1). The average volume of lipoaspirate that was grafted was 23.4 mL (standard deviation [SD] 10.9 mL) and similar between both the groups (*P* > .1). The mean follow-up duration was 7.1 months (SD 3.1 months): 7.2 months, SD 3.5 months in the PWAS group vs 7.0 months, SD 2.2 months in the osmotic filtration group (*P* > .5). The average fat volume retention rate was 73.1% (SD 6.8%) in patients in whom the PWAS was used when compared with 46.1% (SD 5.2%) in patients in whom osmotic filtration was used (*P* > .01).

**Conclusions:**

For patients undergoing low volume fat grafting, the PWAS technology may result in improved fat retention rates when compared with traditional lipoaspirate processing with Telfa.

**Level of Evidence: 4:**

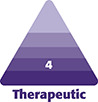

The use of autologous fat grafting as a technique for soft-tissue augmentation and for the correction of contour irregularities is well-established. Fat is often considered a near-ideal soft-tissue filler because of its abundance, easy accessibility, biocompatibility, and lack of immunogenicity.^1^ These factors make it advantageous over commercially available fillers and solid implants, both of which have high costs and the potential for significant complications.^2,3^ Autologous fat grafting has become a standard procedure for both cosmetic and reconstructive purposes throughout the breast and body but has gained particular traction in the craniofacial setting.^4-6^ Although large-volume fat grafting (>100 mL) is often used for breast and body contouring and breast reconstruction, small-volume fat grafting (<100 mL) is primarily used for facial rejuvenation and reconstructive procedures.^7^

Although fat grafting can be an effective and versatile technique, it is plagued by variability in long-term retention, resulting in unpredictable outcomes and repetitive procedures. This topic is well reported in the literature, with a wide variety of retention rates ranging from 26% to 83%.^8^ In a recent systematic review on low-volume facial fat grafting, the pooled fat retention rate was 47% at 3 to 12 months’ follow-up.^8^ Variables influencing fat retention include intrinsic and extrinsic factors. Patient-specific factors such as age, smoking status, genetics, and depot site may influence outcomes. Additional surgeon variables include procedural differences in harvest, processing, injection technique, and postoperative care. Different processing techniques have been described, including gravity separation, filtration, centrifugation, sedimentation, emulsification, and washing, but no single process has been shown to be significantly superior to the others.^9,10^

The poloxamer wash, absorption, mesh filtration system (PWAS) lipoaspirate wash and filtration system (Viality; Sientra, Inc., Irvine, CA) is a novel process that uses proprietary technology to stabilize and concentrate lipoaspirate. It is a self-contained device that efficiently processes lipoaspirate using a poloxamer wash and a mesh filtration system. The poloxamer wash, AuraClens (Sientra, Inc., Irvine, CA), is a nonionic surfactant (active ingredient Poloxamer 188 [P188], 10 mg/mL) that stabilizes the membranes of damaged adipose cells and decreases apoptosis, thus improving cell viability and graft concentration.^11^ Additionally, the absorbent foam layer removes unwanted fluids and maximizes fat concentration, thus improving long-term graft retention.^12^ Figure 1 illustrates a PWAS technology setup in the operating room. Early data from high-volume breast and body cases indicate that the poloxamers, with membrane-sealing capability, can increase graft survival. Specifically, P188 demonstrated a statistically significant decrease in apoptosis, with a resultant increase in graft survival by weight, cell viability, DNA content, and histology.^11^ However, the superior fat retention rates seen with the use of PWAS processing have not yet been reported in low-volume cases.

**Figure 1.**
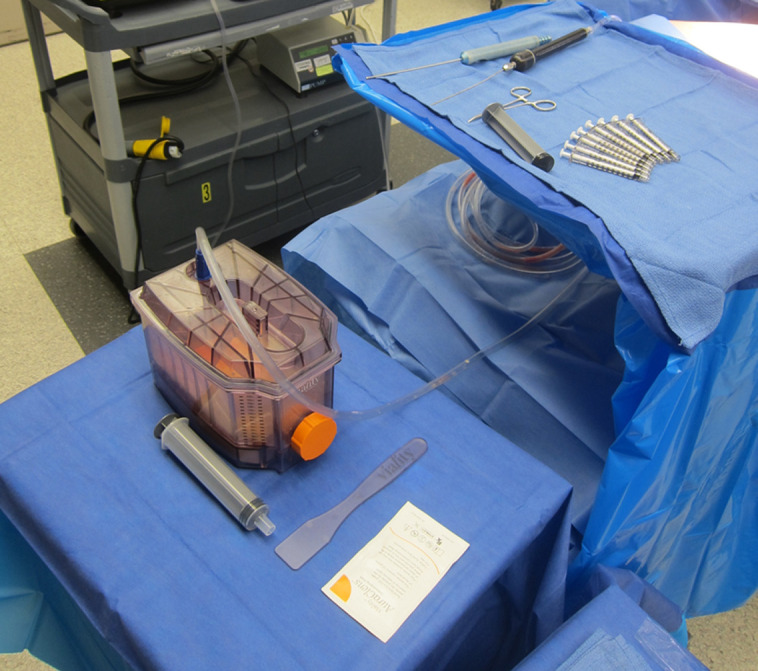
An intraoperative setup of the poloxamer wash, absorption, mesh filtration system technology used for lipoaspirate processing.

The authors of this study aim to build upon these early studies, the authors of which demonstrated success using the PWAS for high-volume fat grafting procedures. We aim to determine whether the PWAS technology could be applied to low-volume fat grafting procedures and improve fat retention when compared with traditional techniques for lipoaspirate processing.

## METHODS

### Study Sample

Medical charts were reviewed to determine the cohort of consecutive patients who underwent autologous fat grafting for facial feminization at a single institution by a single provider between September 2021 and February 2023. The study was reviewed and approved by the Institutional Review Board at Massachusetts General Hospital in Boston, MA (Protocol #2023P002264). All patients included in the study were transgender females over the age of 18. The cost of the procedure was covered by insurance. The inclusion criterion was at least 3 months’ follow-up. The exclusion criteria included the presence of comorbidities that could significantly influence fat retention, including a history of HIV, chemotherapy, radiotherapy, active immune disease, uncontrolled diabetes mellitus, hypertension, chronic use of steroids, connective tissue diseases, chronic blood abnormalities, or other systemic metabolic disorders.

### Operative Techniques

Operative reports from the facial fat grafting procedure were reviewed, and patients were grouped based on the method of lipoaspirate processing. For the purposes of this study, we defined low-volume fat grafting as <50 cc. Options for fat processing included the use of the PWAS or traditional osmotic lipoaspirate filtration. Lipoaspirate was processed using the PWAS according to the manufacturer's recommendations. This included washing with the AuraClens surfactant and filtering and suctioning away debris. The processing method used for each patient was based on surgeon preference and the availability of the PWAS at the time of the operation. Alternatively, osmotic lipoaspirate filtration was accomplished by placing the fat on a sterile nonadherent Telfa sponge and allowing the liquid component to absorb, leaving concentrated fat behind for grafting.

Regardless of the method used for lipoaspirate processing, all fat was harvested and grafted in a similar fashion. Fat was harvested in the operating room under general anesthesia from the anterior lower abdomen utilizing a tumescent technique with nonpower-assisted liposuction. Evidence in the literature suggests that donor sites do not significantly influence the fat retention rate.^13^ The tumescent solution contained 50 mL 0.5% lidocaine, 1 mg epinephrine, and 20 mL of 8.4% sodium bicarbonate per liter of warm saline. After harvesting and processing, an 18 G needle puncture was used to create entry points on the face. Number 4 or 5 Byron cannulas were used to inject fat through 5 mm incisions within relaxed skin tension lines. Fat was injected in 1 to 2 mL aliquots with a Coleman 2 mm blunt tip on a 1 cc syringe until the desired effect was achieved. Injection sites were individualized and compartmental and included anatomic regions such as the hairline, forehead, temple, midface, upper lip, and lower lip. The sites were then gently massaged for ensuring even distribution. Video demonstrates the use of PWAS technology for lipoaspirate processing during a case of facial fat grafting.

### Three-Dimensional Imaging Protocol

All patients had previously obtained preoperative and postoperative 3-dimensional (3D) facial imaging with the VECTRA XT 3D Imaging System (Canfield Scientific, Inc., Fairfield, NJ). Postoperative images were obtained at all routine follow-up appointments. The images were reviewed and compared using the VECTRA Analysis Module (VAM) software (Canfield Scientific, Inc., Fairfield, NJ). Specifically, preoperative baseline images were registered to the 3D axis grid and then compared with subsequent postoperative imaging. Comparative images were viewed side by side, and landmarks were placed in similar locations on each image to register subsequent images to the baseline image. Once complete, the change in facial volume was measured. This was accomplished by selecting the entire facial area to be analyzed on the baseline image and using the VAM software function, which calculates the volume difference between 2 surfaces in parallel projection. The resultant number represents the change in facial volume measured in milliliters. This change in volume can also be appreciated visually by overlaying the 2 images. Areas of white shading represent areas of increased volume. This process was repeated for each preoperative and postoperative comparison.

### Outcome Assessment

Using the above technique, 3D imaging analysis software was used to calculate the change in facial volume between the preoperative and the postoperative images. This volume was then compared with the actual volume injected intraoperatively, as indicated in the operative report. The differences between these values were used to calculate the percent retention, including the SD. The average percent retention between the PWAS and the traditional lipoaspirate processing technique was compared over a range of postoperative time points. A 2-sample *t* test was used to compute the significance between cohorts. Significance was defined as a *P* < .05. Analyses were computed in Stata version 17 (StataCorp LP, College Station, TX).

## RESULTS

### Patient Cohort

During the study period, 11 facial fat grafting procedures were performed using the PWAS and 5 performed using the osmotic filtration with Telfa for lipoaspirate processing. The mean age of the study sample was 46.5 years (SD 17.0; range, 21-70 years) and BMI was 25.3 kg/m^2^ (SD 4.5 kg/m^2^), and they were similar between patients who underwent fat grafting with the PWAS when compared with osmotic filtration (*P* > .1). All patients underwent facial fat grafting alone, except for 1 patient who also underwent rhinoplasty, and 1 control patient who also underwent bilateral brow lift. The mean operative time was 80.7 min (SD 27.9 min) for fat grafting procedures alone and 93.9 min (SD 46.5 min) for those who underwent concurrent rhinoplasty and brow lift. In patients in whom fat grafting was performed alone, the fat was harvested and immediately processed and injected. In the 2 other instances, fat harvesting, processing, and grafting were performed after the rhinoplasty and brow lift. There were no patients in whom fat was left to the open air for longer periods than the standard procedural steps. The mean follow-up duration was 7.1 months (SD 3.1 months) and was similar between both groups (7.2 months, SD 3.5 months vs 7.0 months, SD 2.2 months, *P* > .5). There were no postoperative complications, including no cases of infection, hematoma, seroma, wound dehiscence, or facial nerve palsy. Patient demographics are presented in Table 1.

**Table 1.** Patient Demographics

Variable	Total patients *N* = 26	PWAS patients *n* = 11	Osmotic filtration patients *n* = 5	*P*-value
Age, years, mean (SD)	46.5 (17.0)	45.5 (17.9)	48.6 (18.8)	.751
BMI, kg/m^2^, mean (SD)	25.3 (4.5)	25.9 (5.1)	24.0 (2.4)	.458
Follow-up, months, mean (SD)	7.1 (3.1)	7.2 (3.5)	7.0 (2.2)	.918

PWAS, poloxamer wash, absorption, mesh filtration system.

### Volume Retention

**Figure 2.**
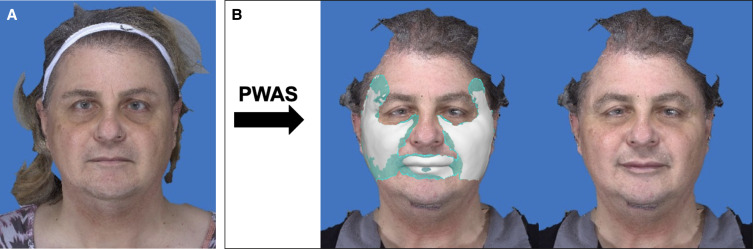
(A) A 60-year-old female patient underwent facial fat grafting of 26 cc to the temples, midface, and lips, for which the poloxamer wash, absorption, mesh filtration system (PWAS) was used. (B) At 12 months’ postprocedure, the retention rate was 61.1%. A three-dimensional imaging analysis shows the volume retained (white shading).

**Figure 3.**
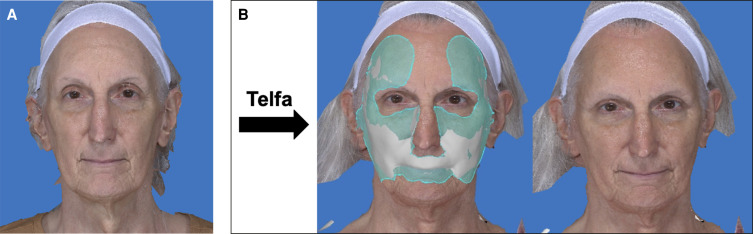
(A) A 68-year-old female patient underwent fat grafting of 32 cc to the temples, midface, and lips, for which osmotic filtration with the Telfa method was used. (B) At 4 months’ postprocedure, the fat retention rate was 52.2%. A three-dimensional imaging analysis shows the volume retained (white shading).

The average volume of processed lipoaspirate injected at the time of surgery was 23.4 mL (SD 10.9 mL) and was similar between both the PWAS group and the Telfa group (26.9 mL, SD 11.0 mL vs 16.9 mL, SD 8.6 mL; *P* > .1). The mean volume retention rate was 73.1% (SD 6.8%) in patients in whom the PWAS was used when compared with 46.1% (SD 5.2%) in patients in whom osmotic filtration was used (*P* < .01). See Tables 2 and 3 as well as Figures 2 and 3 for results. During the reviewed period, no adverse outcomes or complications relating to the fat grafting procedure were noted.

**Table 2.** Poloxamer Wash, Absorption, Mesh Filtration System Cases

Case	Age	BMI	Injected volume (mL)	Measured volume (mL)	% Retained	Follow-up month
Case 1	52	25.8	26.0	22.1	85.0	3
Case 2	21	25.1	33.5	23.1	69.0	3
Case 3	31	21.9	19.0	15.6	82.1	4
Case 4	57	25.9	37.0	26.7	72.2	4
Case 5	29	19.4	16.0	11.5	71.9	5
Case 6	68	25	31.0	24.1	77.8	8
Case 7	70	21.3	15.0	10.1	67.3	8
Case 8	44	27.8	15.0	11.3	75.3	9
Case 9	40	29.4	50.0	36.7	73.4	11
Case 10	29	24.4	21.0	14.5	69.0	12
Case 11	60	38.7	26.0	15.9	61.1	12

**Table 3.** Osmotic Filtration Cases

Case	Age	BMI	Injected volume (mL)	Measured volume (mL)	% Retained	Follow-up month
Case 1	68	23.9	32.0	16.7	52.2	4
Case 2	41	23.7	15.0	7.5	50.0	6
Case 3	28	27.8	10.0	4.2	42.0	7
Case 4	69	21.0	13.5	5.4	40.0	8
Case 5	37	23.7	14.0	6.5	46.4	10

## DISCUSSION

Autologous fat grafting has become a widespread technique in many reconstructive and aesthetic procedures to treat volume and contour defects.^14^ However, unpredictable volume retention remains a significant limitation that often necessitates multiple additional fat grafting procedures to achieve the desired clinical outcome.^4^ Various insults to harvested fat may reduce the clinically relevant volume retained at the recipient site, including cellular debris, oils, blood, tumescent solution, and inflammatory cells and cytokines. Harvested fat is also subjected to nutrient deprivation, as well as mechanical and ischemic stressors, all of which may reduce adipocyte survival.^9^ Additionally, intrinsic factors such as fluid retention and hormone replacement therapies can also affect volume measurements.

Several fat-processing techniques have evolved with the common objective of enhancing adipocyte survival and, consequently, volume retention. Processing techniques have been shown to play an important role in determining the outcomes of fat grafting by modifying the composition, concentration, and volume of the lipoaspirate.^15^ Broadly categorized, the primary processing techniques include sedimentation, centrifugation, washing, and filtration. These techniques are not mutually exclusive and may be combined. However, as of now, there remains a lack of consensus on the optimal fat-processing technique for different fat grafting volumes, because no single technique has consistently demonstrated superior fat graft retention in either preclinical or clinical studies.^8,9^ The current evidence surrounding fat-processing methods remains inconsistent at best, with the authors of several preclinical and clinical studies suggesting superior fat volume retention with washing and filtration devices over other techniques such as centrifugation and sedimentation, and with the authors of other studies offering evidence to the contrary.^1,9,16-19^ Consequently, there has been substantial variability in fat graft processing practice with a reported 56.5% of plastic surgeons using decantation of fat and 43.5% using centrifugation in a recent survey.^20^

In our study, we compared 2 fat-processing techniques in patients undergoing low-volume facial fat grafting for facial feminization: a novel lipoaspirate surfactant wash and filtration system (PWAS) and a traditional osmotic filtration/gauze absorption method. We found that the PWAS technology had an average 73.1% fat retention rate at 7 months postoperatively, which was significantly higher than the 46.1% retention rate observed with the traditional method at a similar follow-up duration. This improvement can amount to significant clinical differences in the context of small-volume fat grafting in the volume-sensitive and well-defined fat compartments of the face. When compared with the current literature, the fat retention achieved with the PWAS technology also surpassed the rates reported by other fat-processing techniques that have been applied in the context of low-volume facial fat grafting and evaluated using imaging technology. Notably, the authors of recent systematic reviews reported a pooled retention rate of 47% at 3 to 12 months’ follow-up, which is lower than the PWAS fat retention rate found in our study at a similar follow–up period, but comparable with the traditional method that we used.^8,9^

Although the precise mechanisms by which the PWAS improves fat retention require further investigation, several factors may be independently or synergistically contributing to this process. The surfactant wash, mesh filter, and absorbable pad system together may allow for optimum mechanical removal of unwanted components of the lipoaspirate, enhancing overall adipocyte viability. The nonionic surfactant polymer (P188) incorporated within the wash may enhance fat processing by maintaining the integrity of cell membranes, thus protecting adipocytes against various types of ischemic, chemical, thermal and mechanical injuries, and apoptosis.^21-23^ Unlike other washing filtration technologies that utilize lactated Ringers within their washing solutions, the dual hydrophobic and hydrophilic properties of the poloxamer, inherent to all surfactants, also confer a soap-like quality that may also contribute to eliminating undesired elements to purify the fat. Our team previously showed a statistically significant improvement of fat graft cell viability with P188 (89%) when compared with saline-treated controls (33%).^11^ Further, the authors of another preclinical study demonstrated significantly greater fat fraction volume from the use of the PWAS technology when compared with a similar washing and filtering system that uses lactated Ringer's solution and no surfactant wash.^12^

To the best of our knowledge, we are the first to compare a novel washing and filtration system to traditional osmotic filtration/gauze absorption methods, which themselves have frequently been reported to achieve superior fat retention and adipocyte viability, and adipose stem cells compared with other techniques such as centrifugation.^13,19,24-28^ Like washing and filtration, gauze techniques have the advantage of causing minimal adipocyte trauma in a time- and cost-efficient manner and, therefore, are commonly employed for low-volume fat grafting procedures. However, as shown in our analysis, gauze techniques may be less effective in generating sufficient fat retention rates in volume-sensitive areas such as the face when compared with novel technologies such as the PWAS. Through the results of the current study, we support the clinical utility of the PWAS technology within the context of low-volume fat grafting, providing evidence of improved fat survival and retention as long as 6 months postoperatively. It is important to acknowledge that successful graft taken is heavily contingent on the harvesting, fat processing, and injection techniques, which often vary among surgeons. Utilization of a standardized closed system device with atraumatic manipulation of fat may be important for achieving long-term graft retention.

Our study had limitations inherent to its design. First, the small sample size and retrospective nature limit the generalizability of the findings as well as the ability to control for relevant confounding variables, highlighting the need for subsequent large prospective studies to corroborate our findings as well as to compare outcomes more broadly between the PWAS and the full spectrum of conventional fat-processing techniques, including methods such as centrifugation and sedimentation. Additionally, our follow-up was limited to 7 months postoperatively. Although analyses of long-term outcomes are needed, achieving longer follow-ups was limited in the current study because control patients underwent subsequent fat grafting with PWAS technology. Further, all patients included in the study underwent feminization procedures and were under estrogen-based hormone therapy at the time of surgery. It is possible that hormone therapy could affect fat retention outcomes; however, this topic has not been extensively researched, and there is currently no consensus on its influence. The homogeneity of our study sample helps mitigate potential confounding factors related to hormone therapy. Surgeon-dependent factors in the fat-transfer technique should also be considered as potential confounders and may greatly affect overall fat retention outcomes. Other critical endpoints, such as patient-reported satisfaction, were not included and may warrant further exploration. Imaging software has inherent limitations because this is an indirect measure of volume change. MRI has been used as an alternative to 3D photography for volumetric analysis, but it is expensive and time-consuming. Furthermore, improper follow-up registration compared with the baseline image can introduce discrepancies in reliable volume analysis. In our cases, a medical assistant captured the images to reduce variability in image capture. Finally, despite the use of 3D facial imaging technology to quantify fat retention, there may remain a degree of uncontrolled operator subjectivity that may limit the reproducibility of reported findings. Despite advancing knowledge of principles associated with fat transfer and widespread clinical adoption, there remains little uniformity in technical aspects of the procedure, which also contributes to heterogeneity in the literature for a direct comparison of outcomes. Continued sharing of knowledge with further refinements in technique may help make fat transfer more reliable.

## CONCLUSIONS

For patients undergoing low-volume fat grafting, PWAS technology may result in improved fat retention rates compared with traditional lipoaspirate processing with Telfa. As facial fat grafting, particularly for the purpose of facial feminization, continues to expand, finding techniques for optimizing outcomes in low-volume fat grafting will be of increasing importance.
